# Recovery of Palladium(II) and Platinum(IV) in Novel Extraction Systems

**DOI:** 10.3390/ma14020285

**Published:** 2021-01-08

**Authors:** Zuzanna Wiecka, Martyna Rzelewska-Piekut, Irmina Wojciechowska, Karolina Wieszczycka, Magdalena Regel-Rosocka

**Affiliations:** Institute of Chemical Technology and Engineering, Poznan University of Technology, ul. Berdychowo 4, 60-965 Poznań, Poland; zuzanna.g.wiecka@doctorate.put.poznan.pl (Z.W.); martyna.rzelewska-piekut@put.poznan.pl (M.R.-P.); irmina.w.wojciechowska@doctorate.put.poznan.pl (I.W.); karolina.wieszczycka@put.poznan.pl (K.W.)

**Keywords:** platinum group metals (PGM), palladium, platinum, chloride solutions, recovery, separation, liquid–liquid extraction, quaternary salts

## Abstract

Recovery of platinum group metals (PGM) from complex aqueous solutions generated as a result of leaching of various spent materials (e.g., spent automotive converters) is a vital issue in the context of the circular economy. In this study pyridinium derivatives containing an imidoamide or imine moiety (i.e., 3-[1-(2-ethylhexyloxyimine)methane]-1-propylpyridinium chloride, 3-[1-(decyloxyimine)methane]-1-propylpyridinium chloride, 3-[1-(decyloxyimine)ethane]-1-propylpyridinium chloride and 4-[1-amine(2-ethylhexyloxyimine)]-1-propylpyridinium chloride) are proposed as novel extractants for recovery of palladium(II) and platinum(IV) from model chloride aqueous solutions. The results of liquid-liquid extraction from one-component solutions of palladium(II) or platinum(IV) showed that quaternary pyridinium salts can be used as effective extractants for platinum metal ions. Moreover, PGM extraction from a two-component mixture proved no evident selectivity in the transfer of one of the metal ions to the organic phase. As the best extractant among the investigated ones, D3EI-PrCl (with straight alkyl chain at substituent) can be pointed out, however, problems with effective stripping or phase disengagement after stripping should be indicated as a drawback of the organic phases used. Further investigation should focus on the improvement of the organic phase properties (e.g., increase in hydrophobicity of the extractants and addition of an organic phase modifier) towards stripping efficiency.

## 1. Introduction

Recovery of platinum group metals (PGM) from spent (secondary) materials, e.g., spent automotive catalytic converters, is considered not only an environmentally important but also economically vital issue. The rapidly growing amount of end-of-life products has become recently a significant resource of precious metals, for example, PGM [1,2]. Spent automotive converters are a more concentrated resource of such valuable metals as palladium and platinum than the natural ores, and, what is important for the circular economy, processing of secondary materials leads to closing a loop of these precious metals.

To dissolve PGM from solid material, chemical dissolution with strongly oxidizing reagents (e.g., aqua regia) is applied. Thus, the pregnant leach solutions after PGM leaching are in chloride or chloride/nitrate media, and the next, very important, step is purification or recovery of PGM from the leach solutions. Extraction of PGM has been investigated from various acidic aqueous solutions by different types of extractants, e.g., hydroxyoximes [3], hydrophobic amines, or quaternary ammonium salts [4,5,6,7,8], pyridinecarboxamides [9], and trialkylphosphine oxides [10]. The separation of various PGMs is based not only on extraction but also on selective stripping [10,11]. Comparison of Pd(II) and Pt(IV) extraction with some commercial extractants is shown in Table 1. More data about Pd(II) and Pt(IV) in various extraction systems can be found, for example, in [5].

Rosocka’s team has developed successful separation of Pd(II), Pt(IV), Ru(III), and Rh(III) from four-component solutions based on two steps of extraction with an ionic liquid solution (trihexyl(tetradecyl)phosphonium chloride, Cyphos IL101) and two steps of stripping with 0.1 M thiourea in 0.5 M HCl followed by 1 M HNO_3_ or 5 M HCl [11]. Truong et al. [10] have proposed separation of Pt(IV), Rh(III) and Fe(III) from the leach liquor of glass industry scraps by extracting Fe(III) and Pt(IV) with solvating extractant Cyanex 923 (a mixture of trialkylphosphine oxides), and then, selective stripping of Pt(IV) from the loaded Cyanex 923 with NaSCN solution. In turn, a mixture of Aliquat 336 (methyltrioctylammonium chloride) and TBP (tributyl phosphate) has been applied for separation of Pt(IV) and Pd(II) from 6 M HCl solution, and in the first stripping step, some amount of Pt(IV) was stripped together with Pd(II) [5]. Further, a solution of 0.7 M thiourea in 0.5 M HCl has been used to strip pure Pt(IV) left in the organic phase. Investigation of PGM extraction with a typical anionic exchanger—Aliquat 336 in various organic diluents—showed that depending on the system, extraction of Pt(IV) or Pd(II) could vary significantly, from less than 10% in Au(III), Pd(II), and Pt(IV) at 0.1 M HCl system to almost 100% in Pt(IV), Rh(III), and Mg(II) at pH 3.4 solution [6,7]. Use of pure ILs based on Aliquat 336 indicated that, on a small laboratory scale, three ionic liquids, i.e., [A336][Cl], [A336][Br] and [A336][I], extracted Au(III), Pd(II) and Pt(IV) quantitatively in most cases; however, the co-extraction of Rh(III) was strongly dependent on the acidity and the chloride concentration [8]. Hence, although many commercial extractants (e.g., Cyanex 923, TBP, Aliquat 336) have been proposed for PGM separation systems, still novel extraction systems are developed to improve the selectivity of PGM separation or decrease the number of extraction-stripping steps. Such novel extractants as piperidine-based [12], phosphonium or imidazolium quaternary salts (ionic liquids) [11,13,14], calixarenes [15,16], crown ethers [17] have been proposed to separate PGM, i.e., Pd(II), Pt(IV), Ru(III), or Rh(III) from aqueous acidic solutions.

In Wieszczycka’s team, the search for advanced new extractants has resulted in the synthesis of pyridine derivatives containing an *N*-alkoxyimidamide or alkaneimine moieties (e.g., *N*-decoxy-1-(pyridin-3-yl)ethaneimine) [18,19], which have been reported to reach very fast equilibrium achieving in the extraction of Zn(II) or Fe(III) from chloride media. Good extraction properties of these derivatives for heavy metals made us investigate the use of quaternary salts of these derivatives, i.e., 3-[1-(2-ethylhexyloxyimine)methane]-1-propylpyridinium chloride, 3-[1-(decyloxyimine)methane ]-1-propylpyridinium chloride, 3-[1-(decyloxyimine)ethane]-1-propylpyridinium chloride and 4-[1-amine(2-ethylhexyloxyimine)]-1-propylpyridinium chloride towards Pd(II) and Pt(IV) recovery from chloride solutions. It is assumed that stable anionic chlorocomplexes which are formed by PGM in chloride solutions should be efficiently extracted by these newly synthesized pyridinium salts. Hence, the novelty of the investigation lies in the use of new extractants in the PGM model system. Based on the results for precursors of these quaternary salts [19] and looking for efficient extractants of PGM ions, it has been assumed that a type of substituent at imine nitrogen and/or the structure of substituent alkyl chain in the studied quaternary salts could influence the efficiency of Pd(II) and Pt(IV) extraction. Thus, in this work are presented preliminary results of the effect of extraction conditions such as hydrochloric acid concentration, type of metal ions, Pd(II) or Pt(IV), the structure of the pyridinium cation, and the extractant concentration.

## 2. Materials and Methods

### 2.1. Reagents and Solutions

One-component model feeds were prepared by dissolving in HCl the required amounts of PtCl_4_ (94%) and PdCl_2_ (99.9%) supplied by Sigma Aldrich (Germany). A two-component model feed containing 1.25 × 10^−3^ M Pt(IV) and 1.25 × 10^−3^ M Pd(II) was prepared by mixing one-component model solutions of Pt(IV), Pd(II). The concentration of H^+^ in the aqueous solutions was determined (702 SM Titrino, Metrohm) by potentiometric titration with 0.1 M NaOH solution.

We had 0.1 M ammonia, 3 M HCl, and 3 M HNO_3_, 0.1 M thiourea in 0.5 M HCl solutions, which were used as stripping phases. All the inorganic chemicals were of analytical grade and were supplied by Chempur, Poland.

**Figure 1:**
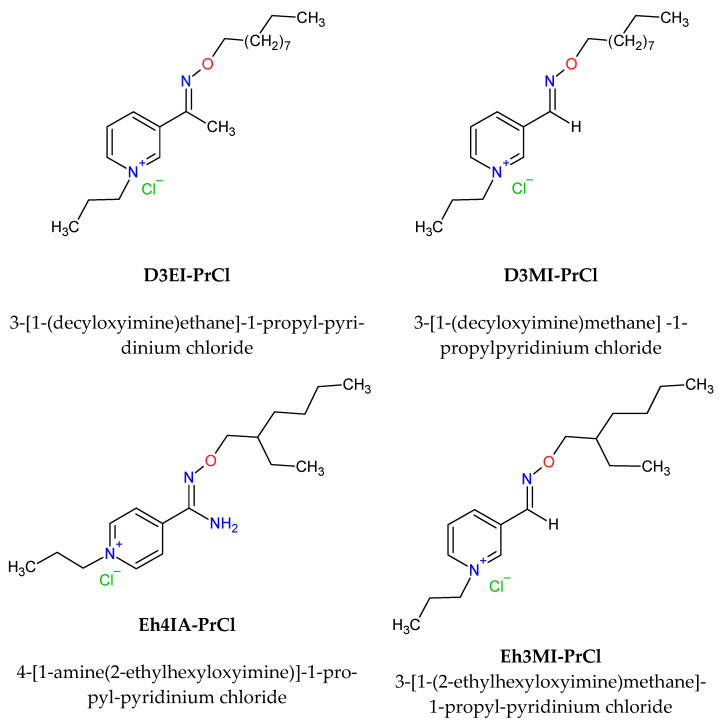
Structures of the quaternary pyridinium salts used as Pd(II) and Pt(IV) extractants.

The organic solutions were prepared by dissolving the required amount of quaternary pyridinium salts: 3-[1-(2-ethylhexyloxyimine)methane]-1-propylpyridinium chloride, 3-[1-(decyloxyimine)methane]-1-propylpyridinium chloride, 3-[1-(decyloxyimine)ethane] -1-propylpyridinium chloride, and 4-[1-amine(2-ethylhexyloxyimine)]-1-propylpyridinium chloride in toluene. Structures of the applied extractants are presented in Figure 1, and the synthesis is described in Section 2.2.

### 2.2. Synthesis of the Extractants

3-[1-(2-ethylhexyloxyimine)methane]-1-propylpyridinium chloride, 3-[1-(decyloxyimine) methane]-1-propylpyridinium chloride, 3-[1-(decyloxyimine)ethane]-1-propylpyridinium chloride, and 4-[1-amine(2-ethylhexyloxyimine)]-1-propylpyridinium chloride were obtained through a two-stage reaction, in which in first stage the appropriate *N*-alkoxy derivatives were obtained by treating pyridine-4-caboximidamide, (pyridin-3-yl)ethan-1-one oxime and oxime of pyridine-3-carboaldehyde with NaOH to form the appropriate oximate and amidoximate salts. Next, the alkylation reaction was carried out at 80 °C using 2-ethylhexyl or decyl bromide and isopropanol as a solvent. The mixture was heated under reflux for 6 h. The crude product was then chromatographed on silica gel with toluene as eluent. The reaction quaternization with propyl chloride was carried out in a round bottom in dry acetone and warm up to 40 °C. Then the mixture was filtered and concentrated under a rotary evaporator. The crude product was recrystallized from acetone. Purities of all compounds were 99.9% and were determined by NMR (^1^H and ^13^C) spectroscopy. All the studied compounds were synthesized with a yield of 96-99%.

3-[1-amine(2-ethylhexyloxyimine)]-1-propylpyridinium chloride (3EhMI-PrCl): ^1^H NMR (400 MHz, CDCl_3_) *δ* in ppm: 0.86 (CH_3_, 3H, t); 1.11 (CH_3_, 3H, t); 1.22–1.39 (14 H, m); 1.56 (CH_2_, 2H, m); 1.69 (CH_2_, 2H, m); 3.61 (CH_2_, 2H, m); 4.17 (O-CH_2_; 2H, t); 7.30 (Hpy-5, 1H, d); 7.94 (Hpy-4, 1H, t); 8.58 (Hpy-6, 1H, s); 8.72 (Hpy-2, 1H, d); 13C NMR (100 MHz, CDCl_3_) δ in ppm: 12.3; 15.1; 24.4; 25.5; 30.0; 32.4; 40.0; 50.05; 62.3 (C-C); 33.9 (CH_3_); 70.6 (C-N^+^); 75.5 (O-C); 123.8 (C = N); 132.9 (C_(Py-5)_); 133.1 (C_(Py-3)_); 145.3 (C_(Py-4)_); 148.7 (C_(Py-6)_); 150.4 (C_(Py-2)_).

3-[1-(decyloxyimine)ethane]-1-propylpyridinium chloride (3DEI-PrCl): ^1^H NMR (400 MHz, CDCl_3_) *δ* in ppm: 0.87 (CH_3_, 3H, t); 1.12 (CH_3_, 3H, t); 1.22–1.39 (14 H, m); 1.80 (CH_2_, 2H, m); 2.20 (CH_2_, 2H, m); 3.76 (CH_2_, 2H, m); 4.33 (O-CH_2_; 2H, t); 7.24 (Hpy-4, 1H, d); 7.90 (Hpy-5, 1H, t); 8.53 (Hpy-6, 1H, s); 8.82 (Hpy-2, 1H, d); 13C NMR (100 MHz, CDCl_3_) δ in ppm: 11.7; 13.8; 22.2; 25.2; 25.5; 29.1; 29.4; 29.6; 30.8 (C-C); 31.9 (CH_3_); 64.6 (C-C-O); 70.6 (C-N^+^); 74.2 (O-C); 121.9 (C = N); 132.5 (C(Py-5)); 135.1 (C(Py-3)); 147.38 (C(Py-4)); 148.2 (C(Py-6)); 150.5 (C(Py-2)).

3-[1-(decyloxyimine)methane]-1-propylpyridinium chloride (3DMI-PrCl): 1H NMR (400 MHz, CDCl_3_) *δ* in ppm: 0.85 (CH_3_, 3H, t); 1.13 (CH_3_, 3H, t); 1.23–1.39 (14 H, m); 1.55 (CH_2_, 2H, m); 1.70 (CH_2_, 2H, m); 3.66 (CH_2_, 2H, m);4.17 (O-CH_2_; 2H, t); 7.30 (H_py-5_, 1H, d); 7.94 (H_py-4_, 1H, t); 8.06 (CHO, 1H, s); 8.58 (H_py-6_, 1H, s); 8.72 (H_py-2_, 1H, d); ^13^C NMR (100 MHz, CDCl_3_) *δ* in ppm: 11.6; 14.1; 22.6; 25.7; 25.8; 29.1; 29.5; 29.6; 31.9; 62.9 (C-C-O); 70.8 (C-N^+^); 75.6 (O-C); 123.6 (C = N); 132.9 (C_(Py-5)_); 133.3 (C_(Py-3)_); 145.1 (C_(Py-4)_); 148.5 (C_(Py-6)_); 150.4 (C_(Py-2)_).

4-[1-amine(2-ethylhexyloxyimine)]-1-propylpyridinium chloride (EH4IA-Cl): 1H NMR (400 MHz, CDCl_3_) *δ* (ppm): 0.93 (CH_3_, 6H, dd); 1.34–1.47 (CH_2_, 8H, m); 1.72 (CH, 1H, ttt); 2.00 (CH_3_,3H, t); 2.75 (CH_2_, 2H, m); 4.07 (OCH_2_, 2H, d); 4,82 (NH_2_, t, 2H); 6.07 (CH_2_; t; 2H); 8.68 (Hpy, d, 2H); 9.01-9.05 (Hpy, d, 2H).13C NMR (100 MHz, CDCl_3_) *δ* in ppm: 12.3; 15.1; 24.4; 25.5; 30.0; 32.4; 40.0; 50.1; 62.3 (C-C); 79.5 (O-C); 93.03 (C-N^+^); 134.1 (Cpy-5); 135.3 (Cpy-4); 143.0 (Cpy-3); 148.5 (Cpy-6); 150.2 (Cpy-2); 164.2 (C=N).

### 2.3. Apparatus

The atomic absorption spectrometer (AAS—ContrAA 300, Analytik Jena) was used for the measurement of metal ion concentrations in aqueous samples at the following wavelengths: 244.8, 266.0 nm for Pd(II) and Pt(IV), respectively.

FT-IR analyses of the organic phases before and after extraction, and after stripping were carried out on a Vertex 70 Spectrometer (Bruker Optics FT-IR) in the range of IR 600–4000 cm^−1^. The spectra of the organic phases were recorded using KRS-5 (Thallium Bromoiodide) cuvette. The spectra were carried out with a standard resolution of 2 cm^−1^.

### 2.4. Extraction and Stripping

Extraction was performed in glass separatory funnels at 21 ± 2 °C for 20 min. The feed solution containing Pd(II) or Pt(IV) or an equimolar mixture of Pt(IV) and Pd(II) in HCl was mechanically shaken with the organic phase (volume ratio A/O = 1) and then allowed to stand for phase separation. Distribution ratio (D) was defined as [20]:

(1) $$D=\frac{{\left[M\right]}_{\left(\mathrm{org}\right)}^{*}}{{\left[M\right]}_{\left(\mathrm{aq}\right)}^{*}}$$

where [M]* means equilibrium metal ion concentration and (org) or (aq) denote the aqueous or the organic phases, respectively. Separation factor (SF) was calculated according to the following equation:

(2) $${\mathrm{SF}}_{\mathrm{Pt}\left(\mathrm{IV}\right)/\mathrm{Pd}\left(\mathrm{II}\right)}=\frac{D_{\mathrm{Pt}\left(\mathrm{IV}\right)}}{D_{\mathrm{Pd}\left(\mathrm{II}\right)}}$$

D_Pd(II)_ and D_Pt(IV)_ are distribution ratios of Pd(II) and Pt(IV), respectively.

Extraction efficiency was defined as percentage extraction (E) [20]:

(3) $$E=\frac{{\left[M\right]}_{\left(\mathrm{org}\right)}^{*}\cdot V_{\left(\mathrm{org}\right)}}{{\left[M\right]}_{\left(\mathrm{aq}\right)}\cdot V_{\left(\mathrm{aq}\right)}}\cdot 100\%$$

where V_(org)_, V_(aq)_ are volumes of the organic and the aqueous phases, respectively, [M]_(aq)_ is the initial concentration of metal ions.

Stripping of Pt(IV) and Pd(II) from the loaded organic phases was carried out in one step for 20 min with various aqueous solutions (A/O = 1). The stripping efficiency (S) was calculated as follows:

(4) $$S=\frac{{\left[M\right]}_{\left(\mathrm{strip}\right)}\cdot V_{\left(\mathrm{strip}\right)}}{{\left[M\right]}_{\left(\mathrm{org}\right)}^{*}\cdot V_{\left(\mathrm{org}\right)}}\cdot 100\%$$

[M]_strip_—metal ion concentration in the aqueous phase after stripping, V_strip_—the volume of the aqueous stripping phase.

Selected experiments with D3EI-PrCl and Eh4IA-PrCl were carried out three times and the error did not exceed 9.5%.

## 3. Results and Discussion

### 3.1. Extraction from One-Component Solutions

PGM, e.g., palladium(II) and platinum(IV), are reported to form in chloride solutions various forms of chlorocomplexes (mainly anionic), differing in the structure and geometry. For example, in acidic solutions (0.1–6 M HCl) containing chloride ions octahedral complexes of Pt(IV) with a coordination number of 6 ([PtCl_6_]^2−^) are reported, while for Pd(II) planar complexes with a coordination number 4 [PdCl_4_]^2−^ are evidenced [21,22]. The presence of PGM anionic species would influence the formation of ionic pairs with quaternary salt extractants, thus, affecting the extraction efficiency of Pd(II) and Pt(IV) from chloride solutions. Therefore, an effect of HCl concentration on Pd(II) and Pt(IV) extraction with the novel pyridinium extractants was investigated in this section.

#### 3.1.1. Extraction of Palladium(II) Species

The effect of HCl concentration on Pd(II) extraction with four different extractants was investigated and is shown in Table 2.

Generally, the salts with the linear (decyl) substituent at oxygen atom, i.e., D3EI-PrCl and D3MI-PrCl, were more effective extractants of palladium(II) species than those with the branched one, i.e., Eh4IA-RCl and Eh3MI-PrCl. Of all the organic salts examined, the D3EI-PrCl compound turned out to be the most effective extractant of Pd(II) chlorocomplexes, extraction exceeded 90% (Table 2). For all the extractants, the percentage of Pd(II) extracted decreased with the rising acidity of the feed. However, the Pd(II) extraction efficiency with Eh4IA-RCl and Eh3MI-PrCl decreased with increasing HCl concentration in the feed more significantly than with decyl substituted salts. It should also be indicated, that the least effective Pd(II) extractant turned out to be Eh3MI-PrCl and the percentage extraction of palladium(II) chlorocomplexes did not reach 50% regardless of HCl concentration in the feed.

Differences in the structure of compounds have a significant impact on the course of extraction. Staszak et al. [23] have examined the interfacial behavior of hydrophobic carboximidamide derivative extractants for Cu(II) extraction. The investigation confirmed that the structural variation of the extractants could not only create a substantial difference in interfacial behavior but could also have a significant effect on the extraction efficiency. It was noted that interfacial tension measurements showed that all the carboximidamide derivatives tested (some of them were precursors of the quaternary salts studied in the current work) at the water/organic phase interface were surface active and adsorbed. It was observed that the major variation in interfacial interaction was caused by a slight structure difference in the alkyl carbon chain, i.e., linear decyl- or branched ethylhexyl substituent. The authors evidenced that the branched-chain carboximidamides in position 3 have the smallest steric hindrance, and thus have easier access to the water/organic interface in the model extraction system that results in better Cu(II) extraction efficiency (a surface extraction mechanism). Although the quaternary salts used as extractants in the present study are derived from some of the compounds studied in [23], their extraction behavior shows that decyl compounds are more efficient, suggesting that these compounds easier access the interface and are likely to be present there in higher concentration. It is opposite to the observations made by Staszak et al. [23], and may result from the ionic nature of the presently studied extractants. Their interfacial properties should be investigated in the future.

It was emphasized in the previous studies [24] on PGM extraction from acidic aqueous solutions with quaternary phosphonium salts that a negative effect of HCl concentration in the feed on the efficiency of Rh(III) or Ru(III) extraction was caused by co-extraction of HCl into the organic phase. The same negative effect of HCl on Ru(III) extraction with Alamine 336 (tertiary amine) and Aliquat 336 (quaternary ammonium salt) was observed by Panigrahi et al. [25]. Therefore, also in the current investigation, HCl concentration before and after Pd(II) extraction was determined in the aqueous phases, and the results of HCl transport to the organic phase are presented in Table 3.

In all the cases, regardless of the used extractant, the transfer of HCl into the organic phase was observed. Co-extraction of H^+^ and Pd(II) species occurred even at the lowest HCl content in the feed solution (0.1 M). The largest amount of HCl is extracted into the organic phase when the feed contains 3 M HCl. Thus, H^+^ co-extraction is likely to affect negatively the Pd(II) extraction from 3 M HCl, and this could be one of the reasons why the extraction efficiency of Pd(II) species with Eh3MI-PrCl does not exceed 50%. On the other hand, comparable amounts of H^+^ are transported to the organic phase containing Eh3MI-PrCl and D3MI-PrCl, while Pd(II) species are much better extracted by D3MI-PrCl than Eh3MI-PrCl. Hence, to summarize, co-extraction of H^+^ to the organic phases studied seems to have no significant influence on Pd(II) extraction.

#### 3.1.2. Extraction of Platinum(IV) Species

Further, for Pt(IV) species extraction with the four pyridinium derivatives, the effect of HCl concentration in the feed was investigated and the results are shown in Table 4.

Similar to Pd(II), the most effective Pt(IV) extractant from the organic salts examined was the D3EI-PrCl, and the Pt(IV) species extraction surpassed 90%. The dependence of the extraction on the concentration of HCl in the feed was opposite to that of palladium and the efficiency of Pt(IV) extraction increased as the acid in the feed increased in concentration. The smallest extraction of Pt(IV) species (below 30%) was obtained for D3MI-PrCl in 0.1 M HCl.

The most effective extractant of Pt(IV) chlorocomplexes was D3EI-PrCl. Regardless of the HCl concentration in the feed, the extraction efficiency of Pt(IV) with D3EI-PrCl reached more than 90%. Contrary to other extractants, the extraction efficiency of Pt(IV) with D3MI-PrCl increases with the increasing acidity of the feed.

Further, as in the case of Pd(II), the concentration of HCl before and after extraction of Pt(IV) was determined in the aqueous phases, and the results of HCl transport to the organic phase are presented in Table 5.

Similarly to Pd(II), the transfer of HCl from the feed into the organic phase by extraction was observed. Hydrogen ion transfer was observed even for the lowest acidity of the feed (0.1 M).

#### 3.1.3. Effect of Extractant Concentration in the Organic Phase

**Figure 2:**
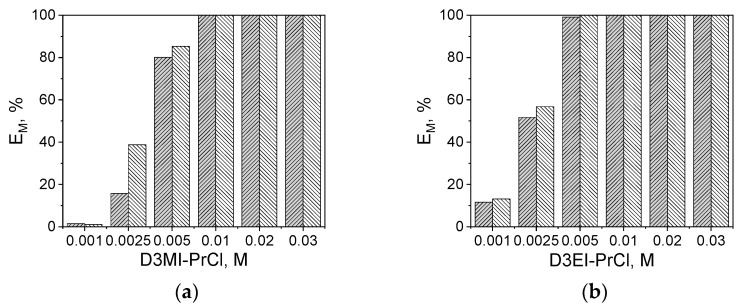
Extraction efficiency of Pd(II) (\\\) or Pt(IV) (///) with (**a**) 5 × 10^−3^ M D3MI-PrCl and (**b**) 5 × 10^−3^ M D3EI-PrCl from feeds containing 2.5 × 10^−3^ M Pd(II) or Pt(IV) in 1 M HCl (A/O = 1).

In order to study the effect of extractant concentration, D3EI-PrCl and D3MI-PrCl salts, which showed the highest efficiency of Pt(IV) and Pd(II) extraction, were selected. The effect of extractant concentration on Pd(II) and Pt(IV) extraction (E and D values) is shown in Figure 2 and Table 6.

Pt(IV) and Pd(II) species were completely extracted into the organic phase when D3EI-PrCl concentration was above 5 × 10^−3^ M (Figure 2b). Likewise, the concentration above 5 × 10^−3^ M of the D3MI-PrCl did not influence the extraction efficiency of Pd(II) and Pt(IV) (Figure 2a). However, a change in the concentration of D3MI-PrCl affected positively the efficiency of metal ion extraction, reaching the quantitative extraction of Pd(II) and Pt(IV) at 0.01 M D3MI-PrCl concentration, which is also visible in high values of distribution ratio (Table 6).

As the most preferred solution is to use the lowest concentration of extractant with the efficiency being the highest, the use of 5 × 10^−3^ M solution of either D3EI-PrCl or D3MI-PrCl seems to be the compromise from the point of view of both extraction efficiency and economic reasons.

### 3.2. Stripping from the Loaded Organic Phases

After loading the organic phases with PGMs during the extraction, the focus was on finding a suitable stripping phase to recover Pd(II) and Pt(IV) species. The different types of extractants were compared, as shown in Table 7.

In most cases shown in Table 7, the loading organic phase and the stripping phase formed stable emulsions. We also found that 0.1 M thiourea in 0.5 M HCl formed stable emulsions with the extractant, while 0.1 M ammonia emulsions separated on the second day. D3EI-PrCl and Eh4IA-PrCl as the extractants of different length and structure of the carbon chain of the substituent were indicated for stripping investigation. For the D3EI-PrCl compound, 0.1 M ammonia was selected as the efficient Pd(II) and Pt(IV) stripping phase. On the contrary, stripping of Pt(IV) and Pd(II) species from the Eh4IA-PrCl organic phase was carried out with two different stripping solutions, i.e., HNO_3_ and HCl, respectively, because 0.1 M ammonia solution and the loaded organic phase formed stable emulsions. The results of Pd(II) and Pt(IV) stripping are shown in Table 8.

Stripping of Pt(IV) from Eh4IA-PrCl is ineffective, while almost 50% of Pd(II) can be stripped from this organic phase with 3 M HNO_3_ solution in one striping step, meaning that at least two steps of stripping are needed to regenerate the organic phase and recover PGM. The highest efficiency of palladium stripping in one step, above 80%, was achieved with the ammonia solution from the organic phase containing D3EI-PrCl. As a result of the stripping with ammonia solution, an unstable emulsion was formed, which prevented the exact separation of the two phases on the same day.

To shortly summarize the results of Pd(II) and Pt(IV) extraction-stripping, a comparison with the commercial extractants presented in Table 1 can be made. Many of the commercial extractants are effective in the extraction of Pd(II) or Pt(IV) species; however, in some cases, two-steps are necessary to extract them efficiently or to extract separately Pd(II) and Pt(IV) [3,5,6]. Even, a well-known commercial extractant—Aliquat 336—is not appropriate for each PGM containing system. It can extract quantitatively Pt(IV) from Rh(III) containing solution (pH 3.4); however, its extraction efficiency from more acidic chloride solutions is much worse (less than 10%) [6,7]. Hence, the authors of this paper see the need to search for new extractants that are effective in acidic solutions. For the results obtained in this study, it should be emphasized that in one step the quantitative extraction of Pd(II) and Pt(IV) species from 1 M HCl is obtained even with as low as 0.01 M of decyl derivatives in the organic phase (D3EI-PrCl or D3MI-PrCl). As the advantage of these new extractants, compared to the commercial ones, can be indicated fast and efficient extraction in one step without the addition of modifiers (e.g., TBP or decanol). However, the formation of a strong chloro-metallate ion with pyridinium as a counterion in the organic phase and, thus, not efficient stripping should be pointed out as a drawback of the proposed pyridine derivatives.

### 3.3. FT-IR Analysis of the Organic Phases

**Figure 3:**
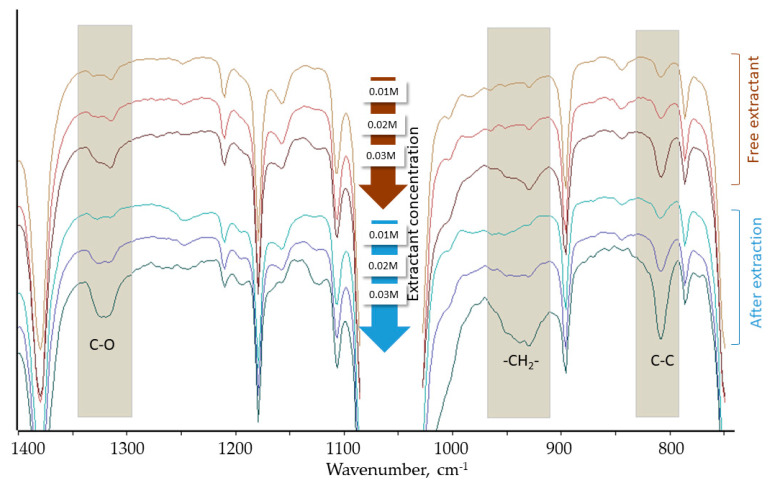
FT-IR spectra of D3EI-PrCl organic phases (0.01, 0.02, and 0.03 M) before and after extraction of Pd(II).

Each organic phase before and after extraction and stripping was analyzed using FT-IR spectroscopy and the spectra are presented in Figure 3.

After the extraction, the changes of vibrational bands of the functional group have been expected, while after stripping the corresponding changes are expected to disappear which confirms regeneration of the extractant. The most interesting results were obtained for D3EI-PrCl and Pd(II) extraction. As can be seen in Figure 3, the FT-IR measurements confirm that Pd(II) is transported to the organic phase due to interaction between the extractant and Pd(II). Even though the signals corresponding to C = C and C = N of pyridine and imine groups are obscured by bands of toluene, the interaction with metals has resulted in changes in other regions of the spectrum. Namely, changes of a vibrational band of C-O (at 1323 cm^−1^) are observed, and the bandwidth widens and, consequently, the band gains a second maximum at 1315 cm^−1^. The increase in the band intensity, as well as band widening, are observed in the region of 910–970 cm^−1^ (vibrations of CH_2_ group), while only an increase in the band intensity is observed at 807 cm^−1^ (C-C vibrations). All the changes are observed both in the case of 0.01 and 0.03 M solution of D3EI-PrCl; however, much higher extractant concentration makes the Pd(II) extraction more efficient and the spectra changes are more visible. Moreover, the study has also shown that ammonia is an effective stripping agent and enables extractant regeneration which is confirmed by the disappearance of spectra changes induced by the extraction.

### 3.4. Extraction from Two-Component Solutions

Extraction from one-component solutions not always gives full information about the separation selectivity of various types of metal ions because their transport can be differentiated by the species existing in the feed or various rates of extraction. Therefore, also extraction from two-component feeds was investigated to verify extraction selectivity of the selected extractants towards Pt(IV) separation.

The preliminary results of extraction from the feed containing both palladium(II) and platinum(IV) species in 1 M HCl are presented in Table 9.

**Figure 4:**
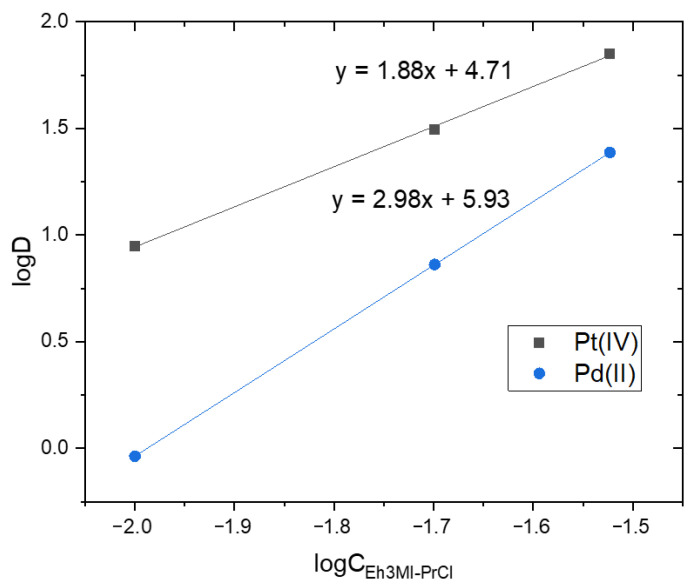
Plots of log D vs. logC_Eh3MI-PrCl_ for Pt(IV) and Pd(II) extraction from two-component feed (A/O = 1, 20 min, feed: 1.25 × 10^−3^ M Pd(II), 1.25 × 10^−3^ M Pt(IV) in 1 M HCl, organics: 1–3 × 10^−2^ M Eh3MI-PrCl in toluene).

It is visible that decyl substituted extractants (D3EI-PrCl, D3MI-PrCl) are not selective to separate species of Pt(IV) from Pd(II) because ions of both metals are extracted almost quantitatively and separation factors (calculated according to Equation (2)) of Pt(IV) separation over Pd(II) are equal to zero. Only the extraction with Eh3MI-PrCl (in the studied concentration range) shows a preference for Pt(IV) species transport to the organic phase compared to Pd(II) transfer. The indicated selectivity could be explained, to some extent, by the number of extractant molecules bound with metal species in the organic phase. Based on the extraction reaction assumed for the investigated novel extractants a slope analysis (Figure 4) can be conducted as a function of the equilibrium concentration of Eh3MI-PrCl to determine the composition of a metal-extractant complex in the organic phase.

The values of slopes presented in Figure 4 suggest that Pt(IV) is extracted by two molecules and Pd(II) by three molecules of the extractant. While ions of both metals are present in the feed they compete for extractant molecules; thus, better extraction of Pt(IV) compared to Pd(II) is likely to result from less extractant necessary to efficiently transfer Pt(IV) from the feed to the organic phase. Although most research on Pd(II) extraction from chloride solutions with quaternary salts indicate that two molecules of the extractant (cations of the salt) form an ion pair with PdCl_4_^2−^ [7,8], hypothetically it is possible (as the steric hindrance of the planar Pd(II) chlorocomplex is smaller than of the octahedral Pt(IV)) that additional molecule could coordinate in the organic phase. The results of these preliminary studies open up a wide field for further research both into establishing the final equation of extraction reaction and improving separation between Pt(IV) and Pd(II).

## 4. Conclusions

Based on the conducted research, it was shown that the proposed novel pyridinium derivatives could be successfully used for the separation of Pt(IV) and Pd(II) species from the aqueous phase employing extraction. It should be emphasized that in one step the quantitative extraction of Pd(II) and Pt(IV) from 1 M HCl is obtained with decyl derivatives (D3EI-PrCl or D3MI-PrCl) in the organic phase. The concentration of HCl in the feed solution significantly affects the efficiency of the extraction of metal ions. At low acid concentrations, Pd(II) species are extracted better than Pt(IV) ones, while Pt(IV) species achieve the highest extraction efficiency at high HCl concentration (3 M). The results of the study partly proved the hypothesis that a type of substituent, i.e., a structural difference in the alkyl carbon chain—linear decyl- or branched ethylhexyl substituent—affected extraction, especially of Pd(II) chlorocomplexes. Reversely, no significant influence of a type of substituent at imine nitrogen was observed what indicates that it is the pyridine nitrogen the plays a crucial role in extracting anionic chlorocomplexes of PGM, not the imine one. As the advantage of these new extractants can be indicated fast and efficient extraction in one step. However, the formation of a strong chloro-metallate ion with pyridinium as counterion in the organic phase and, thus, not efficient stripping should be pointed out as a drawback of the proposed pyridine derivatives. Unlike ammonia, mineral acids as a stripping phase are not as effective. Using two stripping steps could be an alternative. The use of ammonia as the stripping phase has drawbacks, e.g., difficulty in phase disengagement. The separation could be improved by the use of the organic phase additives in the form of appropriate modifiers.

Future research should focus on the selection of a more efficient stripping phase, which ensures also good phase disengagement. Further, the investigation of Pd(II) and Pt(IV) extraction from solutions containing base metals to establish conditions of selective PGM separation from multi-metal solutions.

## Figures and Tables

**Table 1:** Comparison of various Pd(II) and Pt(IV) extraction systems.

Aqueous Feed	Organic Phase	Extraction Efficiency (E, %)	Stripping Efficiency (S, %)	Ref.
Model solutions of Pd(II) or Pt(IV) and real leach solution: 0.39 g/dm^3^ Pt(IV), 0.5 g/dm^3^ Pd(II), 26 g/dm^3^ Fe ions, 20 g/dm^3^ Mn ions, 5.4 g/dm^3^ Ca(II), 6.2 g/dm^3^ Zn(II), 1.3 g/dm^3^ Cu(II), 8.6 g/dm^3^ Al(III), 0.3 g/dm^3^ Ni(II), 0.06 g/dm^3^ Co(II), 0.42 N H^+^	LIX 84I or LIX 64N, or LIX 70, or LIX 984 in dodecane for Pd(II) Alamine 336 in kerosene for Pt(IV)	From leach solution: in I step: E_Pd_~100% with 8% LIX 984 in dodecane at A/O = 5 and pH = 2.75 in II steps: E_Pt_ = 100% with 1% Alamine 336 in kerosene at A/O = 3 and pH = 1	S_Pd_ = 100% Pd(II) stripping with 6 M HCl from the loaded organic phase scrubbed twice with 1.5 M H_2_SO_4_ (to remove Cu(II)) S_Pt_ = 100% with mixture of 1 M NaOH and 1 M NaCl	[3]
120 mg/dm^3^ Pt(IV) and 50 mg/dm^3^ Pd(II) in 1–8 M HCl	Mixture of amines (Aliquat 336/Alamine 336/TOA) and neutral extractants (TBP/TOP/MIBK) in kerosene with decanol addition	E_Pt_ = 100%, E_Pd_ = 38% with 0.01 M Aliquat 336 and 0.4 M TBP in kerosene in 3 steps of counter current extraction at A/O = 1 HCl concentration ↑ (>3 M) = E_Pd_ and E_Pt_ ↓	S_Pd_ = 87 ÷ 100% with 0.001 M thiourea in 0.1 ÷ 0.5 M HCl	[5]
349.4 mg/dm^3^ Pt, 58.9 mg/dm^3^ Rh and 6700.8 mg/dm^3^ Mg, pH 3.4	0.01 M Aliquat 336 in kerosene	Two-step counter current simulation: E_Pt_ = ~100%, E_Rh_ = 0% (A/O = 3.3)	S_Pt_ = ~99.9% with 0.5 M thiourea in 0.5 M HCl (O/A = 6)	[6]
200 mg/dm^3^ of each Au(III), Pt(IV), Pd(II) in a mixture solution of 0.1 M HCl	0.6 g/dm^3^ Aliquat 336 in benzene	E_Au_ > 99%, E_Pt_ = 5%, E_Pd_ = 7% (A/O = 1) pH ↑ (up to 4.5) = E_Pd_ and E_Pt_ ↑	I stripping step: S_Pd_ = S_Pt_ = ~100%, S_Au_ = 0.13% with 1 M HNO_3_ II stripping step: S_Au_ = 100% with 0.05 M thiourea in 0.1 M HCl	[7]
A model solution similar to leachate from end-of-life autocatalyst: 10.5 mg/dm^3^ Pt(IV), 24.0 mg/dm^3^ Pd(II), 6.81 mg/dm^3^ Rh(III) and impurities in 0.001 ÷ 6 M HCl	Pure ionic liquids: [A336][Cl], [A336][Br], [A336][I]	E_Au_ = E_Pt_ = E_Pd_ = 100% with all the ILs HCl concentration ↑ (to 6 M) = E_Rh_ ↓ (to 20%)	I stripping step from [A336][I]: S_Pd_ = 89.8%, S_Rh_ = 4.25%, S_Au_~S_Pt_ < 1% with 1 M NH_4_OH II stripping step from [A336][I]: S_Au_ = 100%, S_Pd_~S_Pt_~S_Rh_ < 10% with 1 M thiourea in 1 M HCl	[8]
1514 mg/dm^3^ Pt(IV) and 178 mg/dm^3^ Rh(III), 1.6 mg/dm^3^ Fe(III) in HCl > 8.5 M	0.05 M Cyanex 923 in kerosene or 0.07 M Cyanex 923 in toluene	E_Pt_ = 85%, E_Fe_ = 100%, E_Rh_ = 0% E_Pt_ = 40%, E_Fe_ = 100%, E_Rh_ = 0%	S_Pt_ = 100%, S_Fe_ = 0% with NaSCN solutions S_Pt_ = 100%, S_Fe_ = up to 60% with HCl solutions	[10]
1.1 mM Pt(IV), 0.8 mM Pd(II), 0.6 mM Ru(III), 0.4 mM Rh(III) in 0.8 M HCl (total Cl^−^ equal to 2.5 M)	0.005 M Cyphos IL 101, Cyphos IL 102, Cyphos IL 104 in toluene	E_Pt_~E_Pd_ > 95%, E_Ru_ = 55%, E_Rh_ < 15% (A/O = 1)	I stripping step from ILs: S_Pd_ = 69 ÷ 98.5% with 0.1 M thiourea in 0.5 M HCl II stripping step from ILs: S_Ru_ = 37.8% with 0.1 M KSCN, S_Pt_ = 69% with 1 M HNO_3_	[11]

↑, ↓—the arrows denote increase and decrease, respectively.

**Table 2:** Values of distribution ratios and extraction efficiency of Pd(II) with 5 × 10^−3^ M Eh4IA-PrCl, D3EI-PrCl, Eh3MI-PrCl, or D3MI-PrCl from aqueous solutions containing 0.1, 1, or 3 M HCl (A/O = 1).

HCl in the Feed, M	D_Pd(II)_			
	After Extraction with Eh4IA-PrCl	After Extraction with D3EI-PrCl	After Extraction with Eh3MI-PrCl	After Extraction with D3MI-PrCl
0.1	5.81	>10^6^	0.940	34.6
1	1.99	>10^6^	0.270	5.82
3	0.81	>10^6^	0.071	6.41
	**E_Pd(II)_, %**			
0.1	83.7	100	48.5	97.2
1	66.5	100	21.4	85.3
3	43.9	96.1	6.60	86.5

**Table 3:** H^+^ co-extraction into the organic phase containing 0.005 M Eh4IA-PrCl, D3EI-PrCl, Eh3MI-PrCl, and D3MI-PrCl (A/O = 1, feed: 2.5 × 10^−3^ M Pd(II) in 0.1–3 M HCl).

HCl in the Feed, M	After Extraction with Eh4IA-PrCl		After Extraction with D3EI-PrCl		After Extraction with Eh3MI-PrCl		After Extraction with D3MI-PrCl	
	H^+^_(aq)_, M	H^+^_(org)_, M	H^+^_(aq)_, M	H^+^_(org)_, M	H^+^_(aq)_, M	H^+^_(org)_, M	H^+^_(aq)_, M	H^+^_(org)_, M
0.1	0.118	0.0065	0.119	0.0055	0.121	0.0035	0.1205	0.0040
1	1.111	0.0170	1.081	0.0470	1.082	0.0460	1.087	0.0410
3	3.112	0.0175	2.995	0.1345	3.043	0.0950	3.045	0.0930

**Table 4:** Values of distribution ratios and extraction efficiency of Pt(IV) with 5 × 10^−3^ M Eh4IA-PrCl, D3EI-PrCl, Eh3MI-PrCl, or D3MI-PrCl from aqueous solutions containing 0.1, 1, or 3 M HCl (A/O = 1).

HCl in the Feed, M	D_Pt(IV)_			
	After Extraction with Eh4IA-PrCl	After Extraction with D3EI-PrCl	After Extraction with Eh3MI-PrCl	After Extraction with D3MI-PrCl
0.1	1.74	13.5	0.949	0.388
1	1.39	>10^6^	0.799	4.02
3	1.03	>10^6^	0.726	6.97
	**E_Pt(IV)_, %**			
0.1	62.2	93.8	48.7	27.9
1	58.0	99.7	44.4	80.1
3	50.4	100	42.0	87.5

**Table 5:** H^+^ co-extraction into the organic phase containing 0.005 M Eh4IA-PrCl, D3EI_PrCl, Eh3MI-PrCl, and D3MI-PrCl (feed: 2.5 × 10^−3^ M Pt(IV) in 0.1–3 M HCl).

HCl in the Feed, M	After Extraction with Eh4IA-PrCl		After Extraction with D3EI-PrCl		After Extraction with Eh3MI-PrCl		After Extraction with D3MI-PrCl	
	H^+^_(aq)_, M	H^+^_(org)_, M	H^+^_(aq)_, M	H^+^_(org)_, M	H^+^_(aq)_, M	H^+^_(org)_, M	H^+^_(aq)_, M	H^+^_(org)_, M
0.1	0.120	0.0015	0.120	0.0015	0.120	0.0015	0.1195	0.0020
1	1.143	0.018	1.035	0.126	1.146	0.0150	1.1155	0.0455
3	3.118	0.058	3.118	0.058	2.974	0.1160	3.0720	0.0180

**Table 6:** Values of distribution ratios of Pd(II) or Pt(IV) with 5 × 10^−3^ M D3MI-PrCl or D3EI-PrCl from feeds containing 2.5 × 10^−3^ M Pd(II) or Pt(IV) in 1 M HCl (A/O = 1).

Extractant Concentration in the Organic Phase, M	D after Extraction with:			
	D3MI-PrCl		D3EI-PrCl	
	Pd(II)	Pt(IV)	Pd(II)	Pt(IV)
0.001	0.011	0.014	0.15	0.13
0.0025	0.63	0.190	1.31	1.10
0.005	5.82	4.02	>10^6^	>10^6^
0.01	>10^6^	>10^6^	>10^6^	>10^6^
0.02	>10^6^	>10^6^	>10^6^	>10^6^
0.03	>10^6^	>10^6^	>10^6^	>10^6^

**Table 7:** Comparison of stripping phases used in the research, (+) correct stripping and good phase disengagement, (+/−) correct stripping with the emulsion disappearing over time, (-) a stable emulsion is formed and (0) no stripping and good phase disengagement.

Stripping Phases	Loaded Organic Phases							
	Eh3MI-PrCl		D3MI-PrCl		D3EI-PrCl		Eh4IA-PrCl	
	Pt(IV)	Pd(II)	Pt(IV)	Pd(II)	Pt(IV)	Pd(II)	Pt(IV)	Pd(II)
0.1 M ammonia	-	-	(+/−)	(+/−)	(+/−)	(+/−)	-	-
3 M HNO_3_	+	-	(+/−)	(+/−)	0	0	+	0
3 M HCl	-	+	-	-	0	0	0	+
0.1 M thiourea in 0.5 M HCl	N/A	N/A	N/A	N/A	-	-	-	-

N/A—data not available.

**Table 8:** The results of Pd(II) and Pt(IV) stripping from the loaded Eh4IA-PrCl and D3EI-PrCl organic solutions.

HCl Concentration in the Feed, M	Percentage Stripping, %			
	Eh4IA-PrCl		D3EI-PrCl	
	3 M HNO_3_	3 M HCl	0.1 M Ammonia	
	Pt(IV)	Pd(II)	Pt(IV)	Pd(II)
0.1	11.8	18.6	15.5	94.6
1.0	6.3	31.5	29.8	89.8
3.0	4.5	46.0	40.2	85.9

**Table 9:** Results of extraction from equimolar mixture of Pd(II) and Pt(IV) with selected extractants (A/O = 1, 20 min, feed:1.25 × 10^−3^ M Pd(II), 1.25 × 10^−3^ M Pt(IV) in 1 M HCl, organics: 5 × 10^−3^ M extractant in toluene).

Extractant Concentration, M	E_Pt(IV)_, %	E_Pd(II)_, %	D_Pt(IV)_	D_Pd(II)_	SF_Pt(IV)/Pd(II)_
Eh3MI-PrCl					
0.03	98.61	96.06	71.0	24.4	2.91
0.02	96.91	87.91	31.3	7.30	4.31
0.01	89.91	47.87	8.90	0.90	9.70
**Eh4IA-PrCl**					
0.03	98.73	99.90	77.7	963	0.08
0.02	98.82	99.37	83.7	158	0.53
0.01	84.12	56.56	5.30	1.30	4.07
**D3EI-PrCl**					
0.03	99.14	100.00	115	1.4 × 10^5^	0.00
0.02	98.88	100.00	88.6	1.4 × 10^5^	0.00
0.01	97.83	100.00	45.0	1.4 × 10^5^	0.00
**D3MI-PrCl**					
0.03	98.63	100.00	72.1	1.4 × 10^5^	0.00
0.02	98.22	100.00	55.2	1.4 × 10^5^	0.00
0.01	98.80	100.00	82.4	1.4 × 10^5^	0.00

## Data Availability

The data presented in this study are available on request from the corresponding author.
